# Clearance of Free Silica in Rat Lungs by Spraying with Chinese Herbal Kombucha

**DOI:** 10.1155/2013/790792

**Published:** 2013-08-19

**Authors:** Nai-fang Fu, Chang-hui Luo, Jun-cai Wu, Yan-yan Zheng, Yong-jin Gan, Jian-an Ling, Heng-qiu Liang, Dan-yu Liang, Jing Xie, Xiao-qin Chen, Xian-jun Li, Rui-hui Pan, Zuo-Xing Chen, Sheng-jun Jiang

**Affiliations:** ^1^Tropical Crops Genetic Resources Institute, Chinese Academy of Tropical Agricultural Sciences, Hainan 571737, China; ^2^Guangxi Zhuang Autonomous Region Institute for Chemical Toxicity Testing, Guangxi Zhuang Autonomous Region Academy for the Prevention and Treatment of Occupational Disease, Guangxi 530021, China

## Abstract

The effects of spraying with kombucha and Chinese herbal kombucha were compared with treatments with tetrandrine in a rat silicosis model. Silica dust (50 mg) was injected into the lungs of rats, which were then treated with one of the experimental treatments for a month. The rats were then killed and the effects of the treatments were evaluated by examining the extent and severity of the histopathological lesions in the animals' lungs, measuring their organ coefficients and lung collagen contents, determining the dry and wet weights of their lungs, and measuring the free silica content of the dried lungs. In addition, lavage was performed on whole lungs taken from selected rats, and the numbers and types of cells in the lavage fluid were counted. The most effective treatment in terms of the ability to reduce lung collagen content and minimize the formation of pulmonary histopathological lesions was tetrandrine treatment, followed by Chinese herbal kombucha and non-Chinese herbal kombucha. However, the lavage fluid cell counts indicated that tetrandrine treatment had severe adverse effects on macrophage viability. This effect was much less pronounced for the kombucha and Chinese herbal kombucha treatments. Moreover, the free silica levels in the lungs of animals treated with Chinese herbal kombucha were significantly lower than those for any other silica-exposed group. These preliminary results indicate that spraying with Chinese herbal kombucha preparations can effectively promote the discharge of silica dust from lung tissues. Chinese herbal kombucha inhalation may thus be a useful new treatment for silicosis and other pneumoconiosis diseases.

## 1. Introduction

Silicosis is a pneumoconiosis disease caused by exposure to various forms of silica dust including crystalline silica and amorphous silica dust [1]. It is caused by the inhalation of fine silica particles, which are deposited in the lungs and ingested by macrophages. This triggers an immune response that stimulates the production of collagen around the particle, resulting in the formation of nodular lesions that obstruct the airways. Occupational exposure to silica dust and the resulting health problems are major public health issues in both developed and developing countries [2]. It was recently reported that 23 million workers in China have been exposed to crystalline silica dust, and that more than ten million workers in India are at risk of exposure [3, 4]. Similarly, more than 1.7 million workers in the USA and more than 3 million workers in Europe are likely to have been exposed to silica dust in the workplace [5, 6]. Silicosis has a range of adverse effects on health, including an increased susceptibility to tuberculosis, lung cancer, and pulmonary heart disease. These problems are exacerbated by the lack of an effective treatment for the condition.

Kombucha is a drink made by fermenting sugar and tea extracts with kombucha. It is rich in acetic acid bacteria, yeast probiotics, acetic acid, and other organic acids that are beneficial to human health and can inhibit the growth of harmful bacteria. It has proven to be a good treatment for atrophic gastritis and gastric ulcer disease and can also help regulate blood pressure, slow aging and prevent and treat various diseases [7]. Kombucha contains two notable groups of microorganisms: *Gluconoacetobacter xylinus *(Xylinum) and yeasts. Xylinum secretes bacterial cellulose through holes in its cell walls. Interestingly, kombucha cultures produce bacterial cellulose more efficiently than cultures of Xylinum alone [8, 9]. The bacterial cellulose produced by kombucha cultures has a number of properties that make it potentially useful in medical applications, including good biocompatibility, thinness (the cellulose sheets are typically only 0.1 microns thick), and a high specific surface area. These properties mean that it functions as a nanoscale functional material with a defined three-dimensional structure and a large number of surface-exposed hydroxyl groups, which allow it to form strong noncovalent bonds with water and also a wide range of ions and organic compounds [10]. It has been demonstrated that bacterial cellulose efficiently adsorbs numerous toxic heavy metal ions, including Cu^2+^, Pb^2+^, Hg^2+^, and Cd^2+^. In addition, it adsorbs a range of nonmetallic toxins such as histamines, ammonia, and NO^2−^ and formaldehyde [11–16]. We therefore hypothesized that if kombucha were sprayed into lung tissues, the bacterial cellulose produced by the culture might adsorb dust and protein precipitates that would otherwise cause the symptoms of silicosis. 

Chinese herbal kombucha preparations can be made by fermenting extracts of various plants with a kombucha culture. If the plants used in these preparations contain biologically active substances, the resulting Chinese herbal kombucha may combine the beneficial effects of the kombucha itself with those of the plants. A range of plants and herbs can be used for this purpose, including licorice, *Siratia grosvenori*, mangosteen, and chrysanthemum. Licorice is known to have numerous pharmacological effects and is widely used in traditional medicine. It contains a range of biologically active compounds, including glycyrrhizin, glycyrrhetinic acid, and the licorice flavonoids. Both licorice and the isolated active compounds are known to have antitussive and expectorant properties. Moreover, it is effective against asthma and offers protection against respiratory pathogens [17, 18]. *Siratia grosvenori* is a specific Chinese plant belonging to the family Cucurbitaceae. When dried, its fruit is very sweet and has a cooling effect. It is an important ingredient of “cooling drinks” and is considered to be beneficial for relieving conditions characterized by high body temperatures, such as inflammation. It is used in traditional Chinese medicine to treat chest pains, dry coughs, sore throats, and aphonia [19]. Mangosteen aqueous extracts have been shown to significantly reduce the frequency of coughing in guinea pigs treated with citric acid or capsaicin and inhibits coughing caused by mechanical stimulation [20]. Chrysanthemum is an important medicinal plant that has various pharmacological effects. Among other things, it functions as an antibacterial, anti-inflammatory, vasodilatory, and antitumor agent, as well as reducing blood pressure and acting as an antioxidant [21]. Teas made from the buds of the camellia plant contain many bioactive compounds, including polyphenols, tea polysaccharides, alkaloids, vitamins, and amino acids, as well as various essential metal ions. As a result, they have a wide range of pharmacological activities, including anti-inflammatory, antioxidant, and antithrombosis effects, as well as being useful in the treatment of diabetes and for reducing blood pressure [22, 23]. 

In light of these facts, we sought to investigate the utility of kombucha and various Chinese herbal kombucha mixtures for the treatment of silicosis and other diseases arising from the presence of dust in the lungs. To this end, rats were exposed to silica dust via tracheal injection and then sprayed with Chinese herbal and non-Chinese herbal kombucha solutions. The results of the kombucha treatments were compared to those observed following treatment with tetrandrine, a compound that is known to be useful for mitigating the symptoms of silicosis [24]. The different treatments were evaluated in terms of their effects on lung anatomy, collagen levels in the lung tissues of the experimental animals, and toxicity.

## 2. Materials and Methods

### 2.1. Materials

Silica dust (99% particle diameter 0.5–10 *μ*m with 80% of particles having diameters of 1–5 *μ*m) was purchased from Sigma Aldrich. Tetrandrine was purchased from the Zhejiang Zhongyi Pharmaceutical Co., Ltd. A kit for measuring collagen (hydroxyproline) levels was purchased from the Nanjing Jiancheng Bioengineering Institute.

### 2.2. Preparation of the Kombucha Mixtures

Kombucha strains were purchased from the Beijing Institute of Food Research.

A kombucha stock solution was prepared according to the method of Ai-jun Lv [23]. The Chinese herbal extract was prepared by mixing tea (0.2% w/w), licorice (0.5% w/w), dried *Siratia grosvenori* fruit (0.5% w/w), and wild chrysanthemum (0.2% w/w) in water and boiling the resulting mixtures for 20 min. The boiled solution was then filtered, cooled to below 30°C, and mixed with a 20% dilution of the kombucha stock solution. After fermenting at 30°C for 2 weeks, the Chinese herbal kombucha solution was considered ready for use in experiments.

### 2.3. Experimental Animals

Test animals were provided by the Hunan Slack Jingda experimental animal company (License number SCXK; Hunan). The animals used were Specific Pathogen Free (SPF) Sprague-Dawley (SD) rats that had been held under quarantine for 5 days.

### 2.4. Tracheal Injection of Method for the Contamination of Silica Dust [25–27]

A 50 mg/mL standard suspension of quartz (silica) dust in saline was prepared. Prior to tracheal injection, samples of the stock solution were autoclaved and then mixed with mycillin (4000 U/mL). The experimental animals were then anaesthetized under sterile conditions and subjected to intratracheal injection with 1 mL of the sterile mycillin-containing silica dust suspension in lungs.

### 2.5. Experimental Groups and Treatments

The 150 experimental animals weighed 180–220 g each and were randomly allocated to five different groups of 30 animals each, 15 males and 15 females (see Table 1).

### 2.6. Measurement of Organ Coefficients and the Wet and Dry Weights of Lung Tissues

At the end of the treatment period, the animals were killed by arterial bloodletting. Their hearts, livers, spleens, kidneys, and other organs and tissues were then immediately removed and weighed, and the organ coefficient for each organ was calculated (organ coefficient = organ weight/body weight × 100%). The trachea and lungs were then removed and separated, and the lungs were stripped of their connective tissue. The connective tissues were soaked in water and the wet lung weight (*M*) was measured. A 0.5 g lung tissue sample was then immersed in acetone for three days for degreasing and then cut into pieces, baked in an oven at 105°C for 12 h, and weighed to determine its dry weight (*m*). The dry weight of the lung was then calculated as 2*m* × *M*.

### 2.7. Determination of Total Lung Collagen (Hydroxyproline) [27]

Lung homogenates were prepared and their hydroxyproline content was determined using the chloramine-T method, according to the instructions provided with the kit. 

### 2.8. Counting and Classification of Cells in Lung Lavage Fluid

The rats were killed by arterial bloodletting from the groin. The trachea was then removed and a V-shaped opening was made at the 1/3 point of the lower trachea. One end of a small plastic hose was fitted with an eight-gauge needle and the other end was inserted into the V-shaped opening and ligated to the lung using fixed lines. 5 mL of saline was taken up in a syringe, which was then affixed to the needle on the end of the tubing. The saline was slowly injected into the alveoli and then slowly withdrawn to yield approximately 3 mL of recovered liquid. The cells within this recovered liquid were counted and classified under a microscope.

### 2.9. Pathological Analysis of Lung Tissues

First, the gross lung morphology was observed. The lung tissue was then fixed using formalin. Conventional paraffin sections were taken and stained with hematoxylin-eosin. The stained sections were then examined using an optical microscope (Olympus BX43). Pathological changes and nodules in the lung tissues were graded, and stained lung tissue collagen fiber and reticular fiber hyperplasia were observed with Model BX43, Olympus Optical microscopy. The results obtained were evaluated according to the diagnostic criteria for pneumoconiosis specified in the national occupational health standards of the People's Republic of China (GBZ25-2002).

### 2.10. Determination of the Free Silica Contents of Whole Lung Samples Using the Pyrophosphate Method [28]

Silica levels in lung samples were measured using the method for determining silica levels in dust samples described in Chinese national standard GBZ/T 192.4-2007 (“Determination of dust in the air of workplace-Part 4: Content of free silica in dust”). Fresh rat lung samples were degreased, dried, and then crushed. Samples of the crushed material (0.10 g) were then analyzed using the previous method.

## 3. Statistical Analysis

All data were recorded in the form *x* ± *s*, where *x* is the mean value from a given number of observations and *s* is the associated standard deviation. The *t*-test was performed using the SPSS software package and used to analyze the significance of differences between groups, using a threshold value of *P* < 0.05.

## 4. Results

### 4.1. Clinical Manifestations of Silica Exposure

In the period immediately following their injection with silica dust, the rats exhibited symptoms of dyspnea, did not eat much, and were sluggish. In the early stages of the treatment period, none of the silica-exposed groups exhibited any significant clinical abnormalities. In the later stages of the treatment period, individual rat weights decreased significantly. Four deaths occurred in total, one in the kombucha treatment group, one in the tetrandrine treatment group, one in the positive control group, and one in the negative control group. No deaths occurred in the Chinese herbal kombucha treatment group.

### 4.2. Changes in Rat Weight following Silica Exposure

The rats were weighed once per week during the treatment period. As can be seen in Table 2, there were no significant differences between the average weights for each group prior to their injection with silica. One week after exposure to the dust, the weight of the negative control group was significantly higher than that of the groups exposed to silica (*P* < 0.01). By the second week of the treatment period, the weights of the rats in the Chinese herbal kombucha and tetrandrine treatment groups were not significantly different to those of the negative control group. The weights of the rats in the kombucha treatment group and the positive control group also increased but remained significantly lower than those of the negative control animals (*P* < 0.05). This indicates that the Chinese herbal kombucha and tetrandrine treatments both promoted the regaining of weight following silica exposure. 

### 4.3. Organ Coefficients after Silica Exposure

At the end of the treatment period, the hearts, livers, spleens, lungs, and kidneys of the rats in each group were removed and weighed, and the corresponding organ coefficients were calculated. The results (Table 3) indicate that there were no significant differences between the negative control group and any of the silica-exposed groups in terms of the organ coefficients for the liver, spleen, or kidney. However, the lung coefficients for all of the dust-exposed groups were significantly greater (*P* < 0.05) than those for the negative control group. There were no significant differences between the lung coefficients for the various silica-exposed groups.

The lung coefficient data shown in Table 3 demonstrate that the Chinese herbal kombucha and tetrandrine treatments inhibited the hyperblastosis of the lung tissue caused by silica exposure. Table 3 also shows that the heart coefficient for the negative control group was significantly lower than those for the positive control group and the tetrandrine treatment group (*P* < 0.05). This suggests that both silica exposure and oral tetrandrine treatment have adverse effects on cardiac health.

However, the heart coefficients for the kombucha and Chinese herbal kombucha treatment groups were significantly lower (*P* < 0.05) than those for the tetrandrine treatment group and were not significantly different from those for the negative control group. This indicates that kombucha and Chinese herbal kombucha are effective at mitigating the cardiotoxic effects of inhaling silica dust.

### 4.4. Cell Counts in Lung Lavage Fluid from Silica-Exposed Rats

The negative control group had the lowest lung lavage fluid cell count (Table 4), averaging 0.308 × 10^9^ cells/mL. Tetrandrine treatment yielded the highest average lavage fluid cell count (7.20 ± 13.62 × 10^9^ cells/mL); one sample from this group had a count of 35 × 10^9^ cells/mL. The cell counts in the lavage fluid from rats in other treatment groups were 2.46 ± 1.78 × 10^9^ cells/mL for the Chinese herbal kombucha group, 1.19 ± 1.04 × 10^9^ cells/mL for the kombucha group, and 1.12 ± 0.75 × 10^9^ cells/mL for the positive control group. Because of the considerable variation within each treatment group, there were no significant between-group differences. 

In general, the greater the total number of cells within the lavage fluid, the more severe the case of silicosis. In conjunction with the finding that tetrandrine treatment suppresses hyperplasia in the lungs (Table 3), the high numbers of cells in the lung lavage fluid of the tetrandrine treatment group indicate that tetrandrine is a poor therapeutic agent due to its toxicity towards the tissues of the lung. The counted cells in the lung lavage fluid for each treatment group were sorted by type (Table 4). It was found that lymphocytes (L), neutral cells (N), and giant divinatory cells (M) accounted for the vast majority of these cells. 

At the end of the treatment period, the most abundant cell type in the lavage fluid of the negative control group was M cells, followed by N and then L cells. Conversely, the lavage fluid of the dust-exposed groups was dominated by N cells, followed by L and then M cells. There were significant differences in the proportions of the different cell types between the negative control group and the silica-exposed groups (*P* < 0.05). M cells are phagocytes that are important for lung health. Their numbers are greatly reduced by dust exposure, causing the relative abundance of N cells to increase.

### 4.5. Hydroxyproline Levels in the Lungs of Silica-Exposed Rats

Hydroxyproline assays were performed on lung samples taken from rats killed one week after the end of the treatment period (30 days after the onset of treatment) and from animals killed three weeks after the end of the treatment period (50 days after the onset of treatment). The results for the day 30 group are shown in Table 5. The only treatment group with hydroxyproline levels that were significantly different to those for the negative control group was treated with non-Chinese herbal kombucha (*P* < 0.05). For the day 50 group, the hydroxyproline levels declined in the following order: positive control > kombucha treatment group > Chinese herbal kombucha treatment > tetrandrine treatment > negative control group. All of the silica-exposed groups other than the tetrandrine treatment group had hydroxyproline levels that were significantly greater than those for the negative control group (*P* < 0.05). Since lung hydroxyproline levels are a biochemical indicator of pulmonary fibrosis, this suggests that tetrandrine is effective at inhibiting fibrosis.

In all cases, the hydroxyproline levels measured for the day 50 animals were substantially greater than those for the day 30 animals (Table 5). Moreover, the between-group differences for the day 50 animals were more significant than those for the day 30 animals. Both of these results indicate that lung fibrosis becomes more severe over time.

### 4.6. Lung Pathology

**Figure 1:**
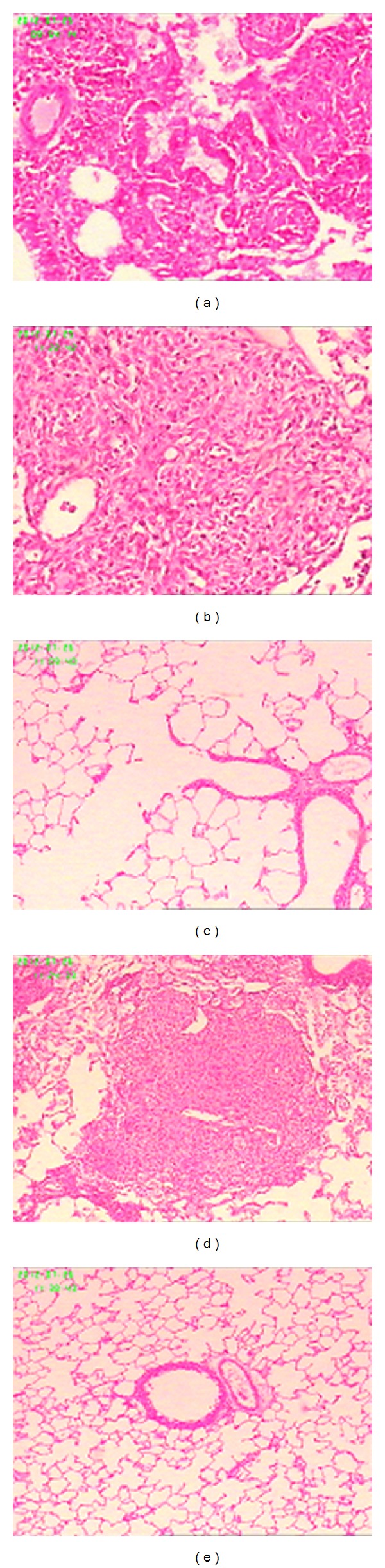
Pathological analysis of lung tissues from rats exposed to silica (400x). (a) Chinese herbal kombucha treatment group; (b) kombucha treatment group; (c) tetrandrine treatment group; (d) positive control group; (e) negative control group.

Visual inspection of the pathological sections of the lungs of the experimental rats (Figures 1(a)–1(e)) indicated that there were no abnormalities among the negative control group. However, significant partial lung consolidation was observed for the kombucha treatment group, the Chinese herbal kombucha treatment group, and the positive control group. The lesions in the sections taken from animals treated with non-Chinese herbal kombucha were particularly heavy, whereas the sections from animals treated with Chinese herbal kombucha were more similar to those for the positive control group. The sections from the tetrandrine treatment group exhibited less extensive lung consolidation and more evidence of lung inflammation.

### 4.7. Wet and Dry Lung Weights

Among the experimental groups, the wet lung weight decreased in the following order: kombucha treatment group > positive control group > Chinese herbal kombucha treatment group > tetrandrine group > negative control group (Table 6). The difference between the values for the tetrandrine treatment group and the Chinese herbal kombucha treatment group was not statistically significant, but that between the values for the positive control group and the tetrandrine group (*P* < 0.05). The dry lung weights for the various groups decreased in the same order as that for the wet lung weights. Based on these results, it was found that the water content of the lungs of rats treated with tetrandrine was relatively low (71.96 ± 0.74%) but that all other groups had similar lung water contents. This implies that tetrandrine treatment causes some level of tissue dehydration. 

Intrapulmonary levels of free silica were measured in lung samples from animals in each of the experimental groups. The highest free silica levels occurred in the tetrandrine treatment group (52.0 ± 12.0 mg). The value for the Chinese herbal kombucha group (38.0 ± 21.0) was significantly (*P* < 0.05) lower than that for both the tetrandrine group and the positive control group (44.0 ± 6.0 mg). Interestingly, the free silica level for the non-Chinese herbal kombucha treatment group was relatively high (54.0 ± 5.0 mg). These results suggest that treatment with Chinese herbal kombucha strongly promotes the discharge of free silica dust, whereas treatment with tetrandrine or non-Chinese herbal kombucha does not promote dust emission and may in fact cause some level of dust enrichment. Over the 30-day treatment period, the rate of silica discharge from the lungs of the rats treated with Chinese herbal kombucha was 0.47 ± 0.69 mg/d (Table 7).

## 5. Discussion

Silica dust is the main pathogenic factor of silicosis. Consequently, the development of effective methods for removing silica dust from the lungs will be essential for effectively treating this disease. This study explored the scope for using Chinese herbal and non-Chinese herbal kombucha preparations as dust-removing probiotic agents for treating silicosis and related conditions. At present, silicosis is treated using drugs such as oxypovidine, tetrandrine, and aluminum citrate, which only alleviate the symptoms of the disease; there is currently no cure. Tetrandrine is the most widely used drug for treating pneumoconiosis in China, and there is strong evidence that it directly or indirectly inhibits collagen gene transcription, thereby reducing collagen synthesis in the affected tissues. Long-term use of tetrandrine can reduce the severity of the respiratory symptoms of silicosis and the number of lung infections suffered, as well as improving lung function. However, it can also cause skin discoloration and itching. Approximately 20% of all patients treated with tetrandrine experience sodium deficiency bloating, and approximately 9.8% experience impaired liver function [29, 30]. The results presented herein suggest that in addition to these effects, tetrandrine may be toxic to cardiac tissue and cause lung dehydration; the latter effect may be related to its known tendency to cause skin dehydration.

Our results indicate that tetrandrine treatment can suppress the formation of collagen in lung tissues. However, the cell counts in lung lavage fluid from tetrandrine-treated rats suggest that it has cytotoxic effects in the lungs and may inhibit the discharge of silica dust. This is consistent with the poor outcomes and severe side effects observed for patients that have been treated with tetrandrine for extended periods of time [29, 30]. As such, it may not be appropriate to use the inhibition of collagen synthesis in lung tissue as the main indicator of effectiveness when evaluating the performance of drugs for the treatment of silicosis. 

Aside from medication, the most common treatment for pneumoconiosis-type diseases such as silicosis is large volume whole lung lavage. This method involves repeatedly flushing the lungs with saline under intravenous anesthesia, together with mechanical ventilation, to remove the pathogenic factor [31]. However, there are several groups of patients that are not suitable for large volume whole lung lavage, including those with (1) conditions that affect blood clotting; (2) severe tracheal or bronchial deformities; (3) illnesses or dysfunctions of the heart, brain, liver, kidneys, or other major organs; (4) cancers or compromised immune systems; (5) active tuberculosis; (6) pulmonary bullae, especially subpleural bullae greater than 2 cm in diameter; (7) severely low pulmonary function; and (8) severe emphysema or related conditions [31]. An analysis of 5000 cases in which large volume whole lung lavage was performed to treat pneumoconiosis or some other lung disorders indicated that the short-term effects (1–3 years) of the treatment are good, but its long-term effects (6–7 years) are not significant. In most cases, lavage causes reductions in chest tightness (reported by 99% of patients), chest pain (reported by 86% of all patients), and shortness of breath (reported by 88% of all patients), with these beneficial effects lasting for around three years. The average amount of dust cleared from each lung was 3,000 to 5,000 mg, including 70–200 mg of free silica. However, extensive removal of pulmonary alveolar macrophages was also observed [32]. While some of the dust and other foreign material is removed from the lungs by lavage, the process can cause significant secondary damage, resulting in complications such as tuberculosis. Additionally, lung lavage is expensive and much of the cost is borne by the patient; since most pneumoconiosis patients have economic difficulties, it would be desirable to find a less costly alternative. 

Our experiments using silica-exposed rats demonstrated that spraying with Chinese herbal kombucha preparations has no toxic side effects and effectively promotes the discharge of silica dust from the lungs. The silica dust exhaust rate for rats (average body weight: 0.200 kg) treated with Chinese herbal kombucha was 0.4 mg/day. Simple linear extrapolation suggests that if a human with a body weight of 65 kg were subjected to the same treatment, the corresponding rate of silica removal would be 130 mg/d. Rats passively accept the aerosol therapy during the test, but a human undergoing treatment would actively inhale the Chinese herbal probiotic. It is therefore possible that the results achieved in clinical trials might be even better than those observed with the rat model. 

The average amount of dust cleared from each lung during lavage is 3000~5000 mg. Based on the results obtained in this work, it would require 23–38 days of spraying with Chinese herbal kombucha to achieve a similar effect. This is consistent with the results obtained when a single pneumoconiosis patient was treated by spraying with Chinese herbal kombucha for three months (see the case report presented in Supplementary Appendix  1 in Supplementary Material available online at http://dx.doi.org/10.1155/2013/790792). The patient experienced significant reductions in the severity of his symptoms within a month, and X-rays taken at the end of the treatment period demonstrated that the treatment had significant beneficial effects on his pulmonary health. Because Chinese herbal kombucha preparations have no toxic side effects and can be also used to treat TB patients and those with cardiopulmonary dysfunctions, they could potentially replace lung lavage as a treatment for pneumoconiosis.

It has been demonstrated that the amount of dust in the lungs of pneumoconiosis sufferers ranges from 0 to 60 grams. Based on the system used to classify cases of pneumoconiosis, first stage cases occur when the lungs contain 0–15 g of dust; this causes dust reticulocyte fibrosis. Second stage cases (15–30 g dust) are characterized by the appearance of mixed nodules due to reticulocyte fibrosis. Third stage cases (30–60 g of dust) are characterized by the appearance of converged fibrosis nodules [33]. Given a dust discharge rate of 130 mg/d, 115 days of treatment with Chinese herbal kombucha would be required to discharge the bulk of the dust in first stage cases, 231 days would be required to treat second stage cases, and 461 days of treatment would be required for third stage cases. A treatment cycle of around one year should thus be sufficient to treat patients with stage I or II pneumoconiosis. However, the lung X-rays in the single-patient case study (see Supplementary Appendix  1) showed that significant quantities of dust were still present within the lungs after three months of treatment, so 2-3 treatment cycles may be required for the complete removal of dust from the lungs in some cases. Even if the treatment only removed dust from the surface tissues of the pulmonary alveolae, significant improvements in lung function would be achieved. However, longer treatment periods may be required to clear deep-seated dust from the lungs.

In this work, two probiotic mixtures were used to treat silicosis. Interestingly, only the Chinese herbal kombucha preparations had unambiguously beneficial effects on dust emission. Non-Chinese herbal kombucha did not promote dust emission, so it appears that the combination of Chinese herbal extracts and the kombucha culture has advantageous synergistic effects. The plant species used to prepare the Chinese herbal kombucha are known to have antitussive, expectorant, and antiasthma function, as well as protecting against respiratory pathogens. Based on these results, they also seem to promote the health of pulmonary tissues and dust emission mediated by the cilia. Kombucha cultures contain two groups of symbiotic microbes: xylinum and yeasts. Xylinum generates bacterial cellulose from the ethanol produced by the yeast; bacterial cellulose is an efficient adsorbing agent that will adhere to dust particles and other substances, thereby facilitating their removal via expectoration.

It should be noted that the treatments examined in this work were only applied over a period of one month. This is relatively short, and it would be desirable to study the effects of treatment with Chinese herbal kombucha over a longer period of time. In addition, the composition of the Chinese herbal kombucha mixture has not been optimized to maximize its therapeutic effect, and it is likely that more extensive research in this area would result in the identification of more potent mixtures. Finally, it would be desirable to determine the precise mechanisms by which treatment with Chinese herbal kombucha promotes the removal of dust from the lungs. Overall, however, the results presented in this work represent the first effective use of a Chinese herbal probiotic to promote the emission of dust from the lungs and the alleviation of inflammation in cases of silicosis. This could have significant consequences for the treatment of silicosis and other pneumoconiosis diseases and more generally for treating conditions involving inflammation of the lungs such as lung protein deposition psychosis. It is very easy to produce and use the Chinese herbal Kombucha. Therefore, the Chinese herbal kombucha would help to globally remove silicosis, pneumoconiosis, and similar diseases in the futures.

## Supplementary Material

Supplementary Appendix 1: A case of gold pneumoconiosis and tuberculosis treated by spraying with Chinese herbal Kombucha.

## Figures and Tables

**Table 1:** Experimental groups and treatments.

Group	Treatment
Chinese herbal kombucha treatment group	Starting four days after being injected with silica, the rats were sprayed with Chinese herbal kombucha. Each rat was sprayed 20 times in the morning and 20 times in the afternoon for five consecutive days per week, over a four-week period.
Kombucha treatment group	Starting four days after being injected with silica, the rats were sprayed with non-Chinese herbal kombucha. Each rat was sprayed 20 times in the morning and in the afternoon for five consecutive days per week, over a four-week period.
Tetrandrine treatment group	The rats were treated with tetrandrine by lavage. Each rat was dosed with 15 mg three times per week for four weeks.
Positive control group	Starting four days after being injected with silica, the rats were sprayed with a 1 g/L solution of NaCl. Each rat was sprayed 20 times in the morning and 20 times in the afternoon for five consecutive days per week, over a four-week period.

Negative control group	Synchronously operated with the positive control group, at the fourth day of instilled with silica. Each rat was sprayed 20 times continuously once in the morning and once in the afternoon for five consecutive days for four weeks.

**Table 2:** Changes in the weight of dust-exposed rats over the course of the experimental period (unit: g).

Treatment	10 days before silica exposure	2 days after treatment	9 days after treatment	16 days after treatment	23 days after treatment
Chinese herbal kombucha	164.0 ± 7.1	202.8 ± 13.9^∆∆^	225.1 ± 18.9	240.1 ± 24.8	253.1 ± 29.3
Kombucha	163.6 ± 6.8	197.1 ± 11.2^∆∆^	222.4 ± 17.9^∆^	238.1 ± 22.0	249.8 ± 29.0
Tetrandrine	164.87 ± 6.2	203.6 ± 12.8^∆∆^	231.7 ± 19.4	246.6 ± 30.0	259.4 ± 32.3
Positive control	165.6 ± 5.9	200.5 ± 12.6^∆∆^	220.8 ± 19.0^∆^	237.2 ± 23.6	248.4 ± 26.7

Negative control	164.7 ± 6.6	217.7 ± 17.8	234.2 ± 23.8	248.7 ± 29.0	263.2 ± 31.1

^∆^Value is significantly different to that for the negative control group (*P* < 0.05).

^∆∆^Value is significantly different to that for the negative control group (*P* < 0.01).

**Table 3:** The influence of the various treatments on the organ coefficients of rats exposed to silica dust.

Treatment	Heart	Liver	Spleen	Kidney	Lung
Chinese herbal kombucha	0.29 ± 0.02*	3.35 ± 0.58	0.25 ± 0.03	0.68 ± 0.05	1.82 ± 0.80^∆^
Kombucha	0.28 ± 0.03*	3.29 ± 0.59	0.25 ± 0.05	0.63 ± 0.05	2.06 ± 0.28^∆^
Tetrandrine	0.33 ± 0.04^∆^	3.64 ± 0.65	0.27 ± 0.05	0.68 ± 0.09	1.46 ± 0.52^∆^
Positive control	0.30 ± 0.02^∗∆^	3.16 ± 0.44	0.26 ± 0.04	0.64 ± 0.07	1.96 ± 0.38^∆^

Negative control	0.28 ± 0.02*	3.34 ± 0.75	0.24 ± 0.05	0.66 ± 0.06	0.80 ± 0.19

*Value differs significantly from that for the tetrandrine treatment group (*P* < 0.05).

^∆^Value differs significantly from that for the negative control group (*P* < 0.05).

**Table 4:** The effects of the various treatments on the cell counts in lung lavage fluid from rats exposed to silica.

Treatment	Total cells (×10^9^/mL)	Cell type (%)		
		N	L	M
Positive control	1.12 ± 0.75	0.685 ± 0.004*	0.247 ± 0.067*	0.068 ± 0.0057*
Negative control	0.31 ± 0.19	0.272 ± 0.226	0.040 ± 0.051	0.692 ± 0.266
Chinese herbal kombucha	2.46 ± 1.78	0.633 ± 0.320*	0.324 ± 0.290*	0.042 ± 0.034*
Kombucha	1.19 ± 1.04	0.718 ± 0.138*	0.256 ± 0.128*	0.027 ± 0.015*

Tetrandrine	7.20 ± 13.62	0.600 ± 0.192*	0.228 ± 0.244*	0.176 ± 0.210*

*Value differs significantly from that for the negative control group (*P* < 0.05).

**Table 5:** The impact of the various treatments on hydroxyproline levels in the lungs of silica-exposed rats.

Treatment	Number of animals	Hydroxyproline level (mg/g*)	
		Day 30	Day 50
Chinese herbal Kombucha	6	0.45 ± 0.18	0.73 ± 0.22^∆^
Kombucha	6	0.50 ± 0.14^∆^	0.75 ± 0.12^∆^
Tetrandrine	6	0.37 ± 0.09	0.56 ± 0.17
Positive control	6	0.45 ± 0.09	0.79 ± 0.28^∆^

Negative control	6	0.34 ± 0.05	0.40 ± 0.06

^∆^Value differs significantly from that for the negative control group (*P* < 0.05).

*Milligrams of hydroxyproline per gram of lung tissue.

**Table 6:** The effects of the tested treatments on wet and dry lung weights in silica-exposed rats.

Treatment	Total wet lung weight (g)	Total dry lung weight (g)	Total lung moisture content (%)
Negative control	1.42 ± 0.43	0.34 ± 0.09	76.15 ± 1.31
Chinese herbal kombucha	3.79 ± 0.93	0.90 ± 0.21	76.14 ± 1.10
Kombucha	4.78 ± 1.14	1.12 ± 0.18	76.19 ± 1.52
Tetrandrine	2.67 ± 0.26	0.75 ± 0.06	71.96 ± 0.74

Positive control	4.38 ± 0.85	1.04 ± 0.14	76.14 ± 1.63

**Table 7:** The effects of the tested treatments on dust removal from the lungs of silica-exposed rats.

Treatment	Whole lung free silica content (mg)	Free silica removed from the lung (mg)	Free silica clearance rate for the whole lung (%)	Silica discharge rate for the whole lung (mg/d)
Negative control	2.0 ± 1.0	—	—	—
Chinese herbal kombucha	38.0 ± 21.0*	13.97 ± 20.65	27.92 ± 41.30	0.47 ± 0.69
Kombucha	54.0 ± 5.0	−2.46 ± 4.68	−4.92 ± 9.36	−0.08 ± 0.16
Tetrandrine	52.0 ± 12.0	−0.18 ± 11.78	−0.35 ± 23.55	−0.01 ± 0.39

Positive control	44.0 ± 6.0	5.81 ± 5.91	11.63 ± 11.83	0.25 ± 0.20

—: because the rats in the negative control group were not exposed to silica, the analysis was not performed in this case.

*Value differs significantly from that for the tetrandrine treatment group (*P* < 0.05).
