# Supplementary material for: Clearance of Free Silica in Rat Lungs by Spraying with Chinese Herbal Kombucha
Source: Evid Based Complement Alternat Med. 2013 Aug 19;2013:790792. doi: 10.1155/2013/790792 (PMC3760100; doi:10.1155/2013/790792)
Supplement: Supplementary file 1 — Supplementary Appendix 1: A case of gold pneumoconiosis and tuberculosis treated by spraying with Chinese herbal Kombucha. [file 790792.f1.pdf]

Appendix 1

A case of gold pneumoconiosis and tuberculosis treated by spraying with Chinese herbal Kombucha

In 2011, I was informed that my brother in law had been diagnosed with pneumoconiosis and tuberculosis, presenting with a cough and serious dyspnea. At the time, he was not able to work. Given the lack of success he had experienced with existing treatments, and after deep and long consideration, I suggested that the Chinese herbal kombucha inhalation as a way of treating his pneumoconiosis. Kombucha contains two symbiotic microorganisms: xylinum and yeast.[1] Xylinum secretes bacterial cellulose from the eyelets of its cell walls. Bacterial cellulose is very effective at adsorbing heavy metal ions and various toxic organic compounds.[2] I therefore hypothesized that if kombucha were administered into the patient's lung, mineral dust and proteinaceous material in the lung would be adsorbed on the cellulose and could then be discharged by expectoration. To further enhance the potentially beneficial effects of the kombucha preparation, it was made using aqueous extracts of health-promoting Chinese herbs (licorice, luohanguo, and chrysanthemum) to yield a substance known as Chinese herbal kombucha. This preparation had previously proven very effective at removing silica

dust from the lungs in a rat model of silicosis. The patient was instructed to spray the Chinese herbal kombucha mixture into his mouth and nose on a daily basis from 2012-2-21 to 2012-5-21, over a period of three months. At the end of this period, the patient reported significant reductions in chest tightness, coughing and other symptoms, and was able to return to work. X-ray images of the patient's chest were acquired before the commencement of the treatment and after its conclusion (see Figure). The treatment reduced the absorption and shadowing caused by lung nodules, made the hilar slightly smaller, and resulted in somewhat less opaque images than those acquired prior to the initiation of treatment. Importantly, the contrast in the non-affected lung tissues was identical in both sets of x-ray images, suggesting that the apparent beneficial effects of Chinese kombucha inhalation. While the x-rays suggest that only a small proportion of the total mineral dust within the patient's lungs was removed, this was apparently sufficient to re-establish effective oxygenation and ventilation, significantly improving lung function. Given that these results were achieved with a relatively short treatment period, it seems reasonable to suggest that more mineral dust might have been removed if the treatment had been extended.

Pneumoconiosis is a serious occupational disease caused by inhaling fine mineral dust. Recent reports indicate that more than 37.7 million workers have been exposed to crystalline silica dust in China, India, the

USA, and Europe. [3] Pneumoconiosis has long been regarded as an incurable disease, and no significant breakthroughs in its treatment have been made during the last decade. [4] The only known effective treatment is whole-lung lavage, which involves washing out mineral dust and other foreign substances from the lungs. However, it is an expensive and painful surgical procedure that cannot be applied to many patients and has a range of potentially adverse consequences. Moreover, it has no beneficial effects in the long term. On average, whole lung lavage removes 6-10g of mineral dust from the patient's lungs.[5] In experiments using a rat silicosis model, treatment with Chinese herbal kombucha resulted in a silica excretion rate of 0.47 mg /d from the lungs. Given that the average bodyweight of the rats in the experiment was 0.2 kg and assuming a body weight of 65 kg for humans, simple linear extrapolation suggests that if the same treatment were applied in humans, the resulting rate of silica/mineral dust excretion would be 152.75mg/d. Thus, treatment with Chinese herbal kombucha treatment for 40--65days should achieve approximately the same level of mineral discharge as can be achieved in a single session of lung lavage. However, the rats would have passively inhaled the kombucha whereas a human patient would presumably actively inhale it. As such, it is possible that the rate of removal may be greater in human patients. Chinese herbal kombucha inhalation is much less costly than existing methods for treating

pneumoconiosis. Together with the promising preliminary results reported herein and those obtained using the rat model, it may be a useful alternative to whole lung lavage and other more invasive medical procedures for the treatment of pneumoconiosis and other lung disorders.

References

1 Greenwalt C J, Steinkraus K H, Ledford R A. Kombucha, the fermented tea: microbiology, composition, and claimed health effects. *J Food Prot* 2000; **63**: 976–81.

2 Chen S, Zou Y, Yan Z, et a1. Carboxymethylated-bacterial cellulose for copper and lead ion removal. *J Hazard Mater* 2009; **161**: 1355-9.

3 Chen W, Liu Y, Wang H,et al. Long-Term Exposure to Silica Dust and Risk of Total and Cause-Specific Mortality in Chinese Workers: A Cohort Study. *PLoS Med* 2012; **9**: e1001206.

4 Centers for Disease Control and Prevention (CDC). Silicosis-related years of potential life lost before age 65 years--United States, 1968-2005.*MMWR Morb Mortal Wkly Rep* 2008; **57** :771-5.

5 Zhang Z, Liu H, Ma G, et al. Clinical analysis of 5000 cases of large volume whole lung lavage treatment of pneumoconiosis and other lung disorders. *Chin J Convalescent Med* 2009; **10**: 956-60 (in Chinese)

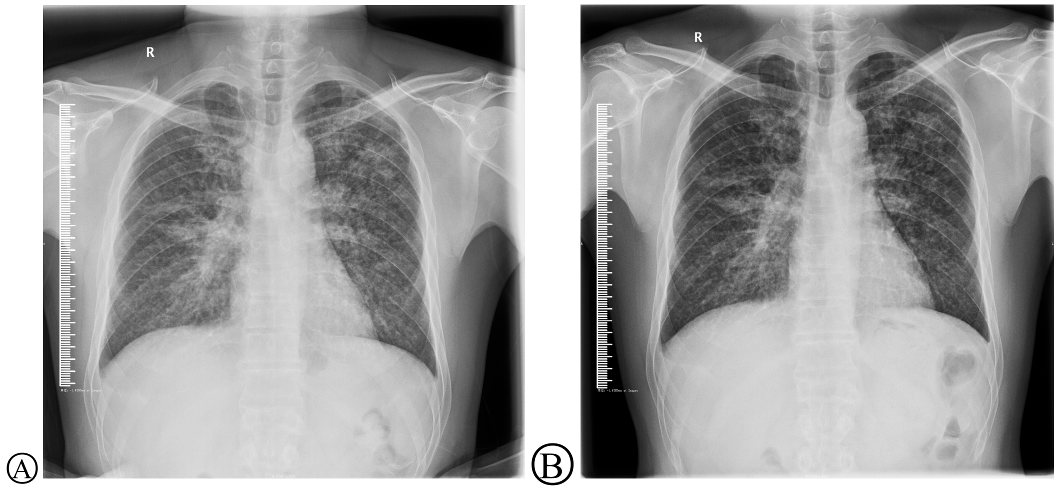

Fig. **A gold pneumoconiosis patient** treated **by spraying with** Chinese herbal **kombucha. A: X-ray taken before treatment. B: X-ray taken after the conclusion of the treatment period.**
